# Leg compliance is required to explain the ground reaction force patterns and speed ranges in different gaits

**DOI:** 10.1098/rsos.250527

**Published:** 2025-10-15

**Authors:** Ali Tehrani Safa, Tirthabir Biswas, Arun Ramakrishnan, Vikas Bhandawat

**Affiliations:** ^1^ School of Biomedical Engineering, Science and Health Systems, Drexel University, Philadelphia, PA, USA; ^2^ Howard Hughes Medical Institute Janelia Research Campus, Ashburn, VA, USA; ^3^ Department of Neurobiology, Northwestern University, Evanston, IL, USA; ^4^ College of Nursing and Health Professionals, Drexel University, Philadelphia, PA, USA

**Keywords:** locomotion, spring-loaded inverted pendulum, walking

## Abstract

Two simple models—vaulting over stiff legs and rebounding over compliant legs—are employed to describe the mechanics of legged locomotion. It is agreed that compliant legs are necessary for describing running, and that leg compliance is also present during walking. Stiff legs continue to be employed to model walking under the assumption that the compliance of the leg during walking is low enough to be considered stiff. Here we study gait choice and walk-to-run transition in a biped with compliance and show that the principles underlying gait choice and transition are completely different from stiff legs. Two findings underpin our conclusions: First, at the same speed, step length and stance duration, multiple gaits that differ in the number of times the leg expands and contracts during a single stance are possible. Among them, humans and other animals choose the (normal) gait with M-shaped vertical ground reaction forces (vGRF) not just because of energy considerations but also constraints from forces. Second, the transition from walking to running occurs because of three factors: vGRF minimum at mid-stance characteristic of normal walking, synchronization of horizontal and vertical motions during single support, and velocity redirection during the double support. The insight above required an analytical approximation of the double spring-loaded pendulum (DSLIP) model describing the intricate oscillatory dynamics that relate single and double support phases. Additionally, we also examined DSLIP as a quantitative model for locomotion and conclude that DSLIP speed range is limited. However, insights gleaned from the analytical treatment of DSLIP are general and will inform the construction of more accurate models of walking.

## 1. Introduction

The center of mass (CoM) movement and the forces exerted on them follow relatively simple patterns conserved across animals, suggesting that the overall animal–substrate interactions and, therefore, the underlying mechanical principles are simple and general [1,2]. The best example of this generality is observed during running: irrespective of the size of the animal and the number of legs it uses during running, the CoM reaches its minimum height at mid-stance, and the vertical ground reaction force (vGRF) has an inverted ‘U’-shaped profile with a mid-stance maximum. This profile is well-explained by the spring-loaded inverted pendulum (SLIP), in which the mass of the animal is concentrated at a point and supported by a massless spring [1,3–8].

Unlike running, it is unclear whether leg compliance is important for walking—the gait used at low speeds. Initially, the inverted pendulum (IP) model, which uses a non-compliant or rigid leg, was used to model walking [9–11]. The IP model successfully models the energetics of walking [12–15], explaining correctly the exchange of kinetic and potential energy during walking: during the first half of the stance phase, the speed of the CoM decreases and height increases; the increased potential energy is reconverted into kinetic energy during the second half of the stance phase.

With modifications, IP can also model the work done during the step-to-step transition. During walking, the CoM velocity vector is directed downward at the end of the step and must be redirected upward before the next step [12,13,16]. In the IP model, velocity redirection occurs instantaneously; therefore, the work performed during the transition cannot be estimated. Regardless, many trends in the work performed during walking can be explained by distributing the force impulse in IP over a finite period of time; however, these modifications are entirely ad hoc. Another, perhaps more fundamental limitation of the IP model is that it cannot model the double-humped or M-shaped vGRF during walking. This limitation has been addressed in many ways: by modelling non-impulsive impact forces at the beginning and end of each step and by using a telescoping actuator with bounds on impact forces [17,18]. However, the model that produces the most naturalistic force profiles assumes a linear relation between force and leg length, implying that a linear spring is probably necessary to model vGRF during walking. This limitation—as shown in this study—is a significant flaw when considering the walk–run transition. *It turns out that the mid-stance minimum, while making M-shaped walking an economical gait, is also a reason why the range of walking speeds is limited, and this is a key new insight we provide by including spring compliance*.

**Figure 1.**
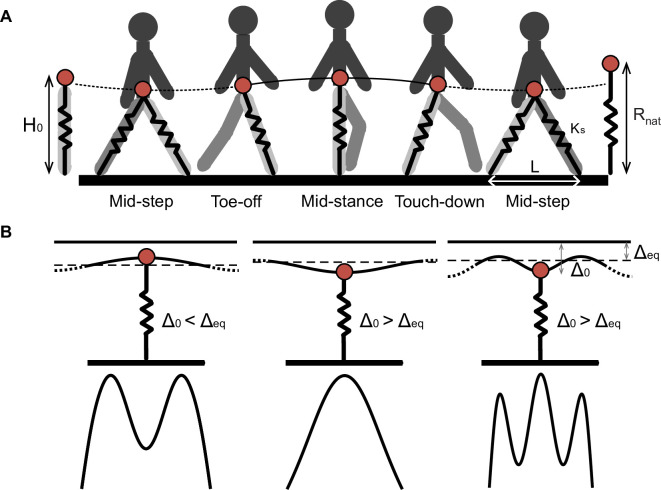
The position of the CoM at mid-stance in relation to the equilibrium point of the spring-mass system determines the GRF profile. (A) Gait cycle for normal human walking showing the mid-stance maximum in height and a mid-step minimum in height. The DSLIP model is overlayed on the step cycle. Solid lines and dotted lines represent single- and double-stance phases, respectively. (B) The top row shows the position of the CoM at mid-stance in relation to the equilibrium position. During human walking (left), the CoM at mid-stance is above this equilibrium point; the resulting vGRF will be at its minimum and produce an M-shaped vGRF. In grounded running (middle), the CoM is at its minimum height below the equilibrium point, resulting in the maximum spring contraction/force at mid-stance. Walking with multiple oscillations (right) can have either a maximum or minimum CoM height regarding the number of oscillations. Again with the same logic, the extremums of the vGRF profile are defined based on the position of the CoM related to the equilibrium point.

The limitation of the IP model and the recent realization that legs are compliant during walking [11,19] led to the development of the double SLIP (DSLIP) model, in which each leg of a biped is modelled as a spring (figure 1A). DSLIP extends SLIP with a double-stance phase during which CoM is supported by two ‘springy’ legs [2,20]. DSLIP can produce M-shaped ground reaction forces (GRFs) observed during human walking by providing smooth velocity redirection during the double-stance phase. It also produces trajectories with mid-stance heights that are lower than IP and more in accordance with experimental data. Although DSLIP is an attractive model, there are several issues regarding DSLIP as a model. The first issue is whether DSLIP can explain the choice of gait at a given speed. Although it is clear that DSLIP is versatile and all the major gaits observed during bipedal walking can emerge from the DSLIP model [21], the speed range over which DSLIP supports M-shaped walking is limited compared with the range observed in animals [2,22]. The reasons for this limited range of speed supported by DSLIP are not understood. It is unclear whether this disparity in speed ranges is a fundamental limitation of the DSLIP model or a matter of quantitative detail.

A second related issue is that of gait transition from M-shaped walking. The issue of what mechanical principles underlie gait transitions in a DSLIP model or in a system with compliant legs has not been studied well enough [18], and it is assumed that the reasons for gait transitions in DSLIP are the same as those in the IP model because the effective spring during walking is stiff. This assumption seems reasonable at face value, but has never been rigorously examined. In this study, we will show that the mechanical principles underlying the gait transition are completely different when compliance is added to the leg.

A third issue is how well DSLIP models the kinematics and mechanics of human walking. This question has not been evaluated rigorously. In studies in which DSLIP is compared with experimental data, it predicts within-step variations in CoM height and GRFs [22,23] that are larger than those observed experimentally. A larger issue is how a successful model is defined. Most studies focus on a single aspect of locomotion, such as GRFs. Considering GRFs, CoM kinematics and real non-dimensionalized time (and not normalized time) at the same time is crucial because the CoM height, H, along with the gravitational acceleration constant, g≈9.8 m s^−^${,}^{2}$, determines the natural timescale of the system $\sqrt{\frac{R_{nat}}{g}}$, where $R_{nat}\approx H$ is the natural spring length. A successful model must produce realistic GRFs within the constraints of experimentally observed CoM kinematics and stance duration. These three constraints are rarely satisfied [22,24,25] simultaneously in most studies of locomotion, leaving the problem under-constrained. A previous study used this approach to model the single-support phase of human walking [26].

These issues raise the question of whether adding compliance to the leg is worth the added complexity. In this study, we show that adding leg compliance through the DSLIP model is worth the complexity. During locomotion, the radial and angular motions of the CoM must be synchronized. Leg compliance provides a natural mechanistic basis for understanding the implications of this synchronization. We show that leg compliance explains the gaits observed at a given speed and how they relate to different oscillatory modes of the spring. *We further argue that the normal gait with the characteristic ‘M’-shaped GRF is preferred over other available walking gaits with different number of oscillatory cycles because of a combination of energy and force constraints. The other gaits either require more energy or unusually large forces, and this is another key insight of our work*. Surprisingly, the preference for the M-shaped vGRF also limits the range of speed over which walking is possible by requiring a large redirection of the velocity vector while simultaneously making it difficult to extract the required vertical forces necessary for the same. Thus, the reasons why a walker with compliant legs undergoes a gait transition are fundamentally different from those for a stiff-legged walker. While the DSLIP model is particularly limited in its ability (probably because springs are assumed to be linear) to produce M-shaped GRFs, it does explain the fundamental reason why humans (and other animals) transition to faster gaits at size-specific speeds (Froude number $\equiv (\mathrm{speed}{)}^{2}/gR_{nat}$) that are significantly lower than 1, the approximate transition speed predicted by IP.

## 2. Results

Throughout the manuscript, we employ the DSLIP model (figure 1A) in which both legs are modelled as massless springs with the same stiffness , $K_{s}$, and natural length, $R_{\mathrm{nat}}$. The dynamics of the single-stance phase are the same as SLIP; the swing dynamics are not modelled. The single-stance phase transitions to a double-stance phase when the distance between the CoM and the future footstep equals the spring’s natural length, and we assume that the swing leg has ‘touched down’. We will focus on symmetric gaits so that the lift-off of the receding leg and the touch-down of the leading leg occur at time points given by time-reversal symmetry about the mid-step time. All variables in their dimensional and dimensionless forms are enumerated in the table below (table 1).

**Table 1.** The model’s parameters.

parameter	symbol	dimensionless form	relation
mass	*M*	N/A	N/A
acceleration of gravity	*g*	N/A	N/A
weight	*W*	N/A	*W* = Mg
natural spring length	$R_{nat}$	N/A	N/A
time	t	τ	$\tau =t\sqrt{\frac{g}{R_{nat}}}$
single-stance time	$t_{s}$	$\tau_{s}$	$\tau_{s}=t_{s}\sqrt{\frac{g}{R_{nat}}}$
double-stance time	$t_{d}$	$\tau_{d}$	$\tau_{d}=t_{d}\sqrt{\frac{g}{R_{nat}}}$
spring stiffness	$K_{s}$	γ	$\gamma =\frac{K_{s}R_{nat}}{W}$
step length	*L*	λ	$\lambda =\frac{L}{R_{nat}}$
radial coordinate	R	r	$r=\frac{R}{R_{nat}}$
initial radial coordinate	$R_{0}$	$r_{0}$	$r_{0}=\frac{R_{0}}{R_{nat}}$
radial coordinate at equilibrium	$R_{eq}$	$r_{eq}$	$r_{eq}$ = 1 − $\frac{1}{\gamma }$
radial coordinate at transition	$R_{⋆}$	$r_{⋆}$	$r_{⋆}=\frac{R_{⋆}}{R_{nat}}$
height of CoM	H	h	$h=\frac{H}{R_{nat}}$
height of CoM at mid-stance	$H_{0}$	$h_{0}$	$h_{0}=\frac{H_{0}}{R_{nat}}$
spring contraction	Δ	δ	δ=1−r
initial spring contraction	$\Delta_{0}$	$\delta_{0}$	$\delta_{0}=1-r_{0}$
spring contraction at equilibrium	$\Delta_{eq}$	$\delta_{eq}$	$\delta_{eq}$ = $\frac{1}{\gamma }$
spring contraction at transition	$\Delta_{⋆}$	$\delta_{⋆}$	$\delta_{⋆}=1-r_{⋆}$
angular coordinate	θ	θ	N/A
angular coordinate at transition	$\theta_{⋆}$	$\theta_{⋆}$	N/A
initial angular velocity	${\dot{\theta }}_{0}$	$\Omega_{0}$	$\Omega_{0}={\dot{\theta }}_{0}\sqrt{\frac{R_{nat}}{g}}$
maximal spring energy	E	ϵ	$\epsilon =\frac{E}{MgR_{nat}}=\frac{1}{2}\gamma \delta_{\max}^{2}$
oscillation frequency	N/A	ω	$\omega =\sqrt{\gamma }$
oscillation phase	N/A	ϕ	ϕ=ωt
oscillation phase at transition	N/A	$\phi_{⋆}$	N/A
horizontal displacement of the CoM	$X_{com}$	x	$x=\frac{X_{com}}{R_{nat}}$
vertical displacement of the CoM	$Y_{com}$	y	$y=\frac{Y_{com}}{R_{nat}}$
average CoM's speed	$V_{com}$	v	$v=\frac{V_{com}}{\sqrt{gR_{nat}}}$
Froude number	N/A	Fr	$Fr={\left(\frac{\lambda }{\tau_{s}+\tau_{d}}\right)}^{2}$
horizontal velocity at transition	N/A	$v_{x_{⋆}}$	$v_{x_{⋆}}\approx (1-\delta_{⋆})\Omega_{0}\cos\;\theta_{⋆}$
vertical velocity at transition	N/A	$v_{y_{⋆}}$	$v_{y_{⋆}}\approx -(1-\delta_{⋆})\Omega_{0}\sin\;\theta_{⋆}$
vertical acceleration at transition	N/A	$a_{y_{⋆}}$	$a_{y_{⋆}}\approx \gamma \delta_{⋆}\cos\;\theta_{⋆}-1$

### 2.1. Emergence of different walking gaits and their energetics

#### 2.1.1. Different gaits are oscillatory modes of the double spring-loaded inverted pendulum model

The DSLIP model can function in multiple modes [2,21,27–29]; these modes include common modes of animal locomotion. These different modes arise from different positions of the CoM in relation to the equilibrium length of the spring (figure 1). To describe the different modes, it is convenient to use the spring compression, $\Delta \equiv R-R_{nat}$. Each mode is an oscillation around the fixed point, $R=R_{eq}=R_{nat}-\Delta_{eq}$, of the spring-mass system given by the Δ where the spring force balances gravity, $\Delta_{eq}=Mg/K_{s}$, where M is the mass of the subject. Assuming symmetry, at mid-stance, the radial coordinate and the height must be either at a maximum or a minimum. At the take-off point, the leg reaches its maximal length or the natural length, $R_{\mathrm{nat}}$. Whether the mid-stance height is at a maximum or a minimum is determined by the relationship between the compression at mid-stance, $\Delta_{0}$, and $\Delta_{eq}$: if $\Delta_{0}>\Delta_{eq}$, the weight is larger than the spring force at mid-stance, the net vertical force points downwards, the second derivative of the height at mid-stance, ${\ddot{H}}_{0}$, is negative and the CoM must go down, resulting in a maximum in height and leg length. Thereafter, it must undergo approximately an integral number of oscillations before take-off. Normal human walking with its mid-stance height maxima is the most common gait of this kind, with approximately a single radial oscillation between the mid-stance and take-off (figure 1B, left).

In contrast, if the leg starts below the equilibrium, $\Delta_{0}<\Delta_{eq}$, the spring force is larger than the weight, net vertical force is upward, $\ddot{H_{0}}>0$, resulting in a height and leg length minima. The radial coordinate undergoes approximately half-integral oscillations before take-off (figure 1B, middle). The lowest oscillatory mode with approximately half of an oscillation corresponds to the grounded running gait that is employed over a limited speed range in humans but over a large range of speed in some birds [27,30,31]. In figure 1B, right, we also show gait patterns of this type with more than one vertical oscillation. Gaits with more than one oscillation are found infrequently and are sometimes classified as a walking gait when there is a mid-stance minimum in the GRF or as running when there is a mid-stance maximum [27].

**Figure 2.**
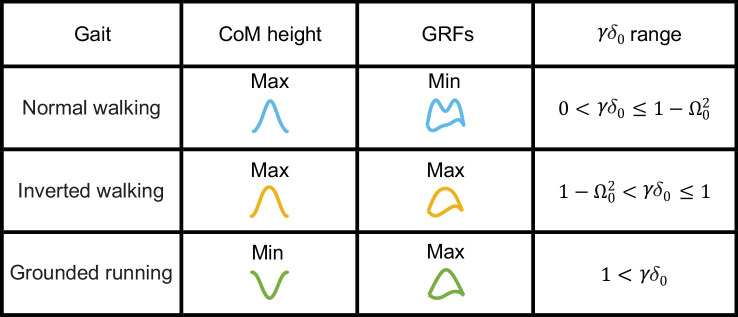
vGRFs and CoM trajectories for different gaits with at most a single contraction–expansion cycle between mid-stance and mid-step and the limits within which each is supposed to occur. The range over which different gaits are observed depends mostly on whether the spring is compressed more or less than the compression necessary to balance the gravitation force. The $\Omega_{0}^{2}$ term compensates for the centripetal acceleration and will be small for most walking speeds. See electronic supplementary material, appendix B for a detailed derivation.

The gait patterns and the ranges over which they are found, when we have at most one oscillation, are summarized in figure 2 in non-dimensional units. We only consider gaits with one oscillation because they represent the most commonly found gaits. Due to the centrifugal force resulting from the angular motion, gait transition occurs at a CoM height, $H_{0}$, that is slightly higher than the equilibrium height (see electronic supplementary material, appendix B for a detailed derivation). Due to the centrifugal force, there is a small range of $\Delta_{0}$ values for which the gait has a mid-stance maximum in height without an M-shaped GRF (inverted walking). Finally, there is a large range of values where grounded running, with a height minimum and inverted ‘U’-shaped vGRF maximum, is observed, consistent with previous work [27,31,32]. The grounded running and inverted walking gaits are inverted gaits owing to their inverted ‘U’-shaped vGRF maximum.

#### 2.1.2. Gait parameter space

To evaluate the exact ranges, we found limit cycle solutions. *A priori*, there are five dimensional parameters that control the evolution of a symmetric gait: stiffness and natural length of the leg spring, $K_{s}$ and $R_{\mathrm{nat}}$, respectively, the step length, L, and the height and angular velocity at mid-stance, $H_{0}$ and ${\dot{\theta }}_{0}$, respectively. Time-reversal symmetry requires that at mid-stance and mid-step, $\dot{H}$*,* must be zero, which provides an additional constraint, leaving only four independent parameters among $\left\{K_{s},R_{\mathrm{nat}},L,H_{0},{\dot{\theta }}_{0}\right\}$, that parametrizes limit cycles. To simplify the analysis further, we used dimensionless quantities (by setting $R_{\mathrm{nat}}=1$): the dimensionless angular speed and length contraction at the mid-stance, $\Omega_{0}$ and $\delta_{0}$, the dimensionless spring constant, γ, and relative step length, λ. Of these four, only three are independent due to the limit cycle requirement.

**Figure 3.**
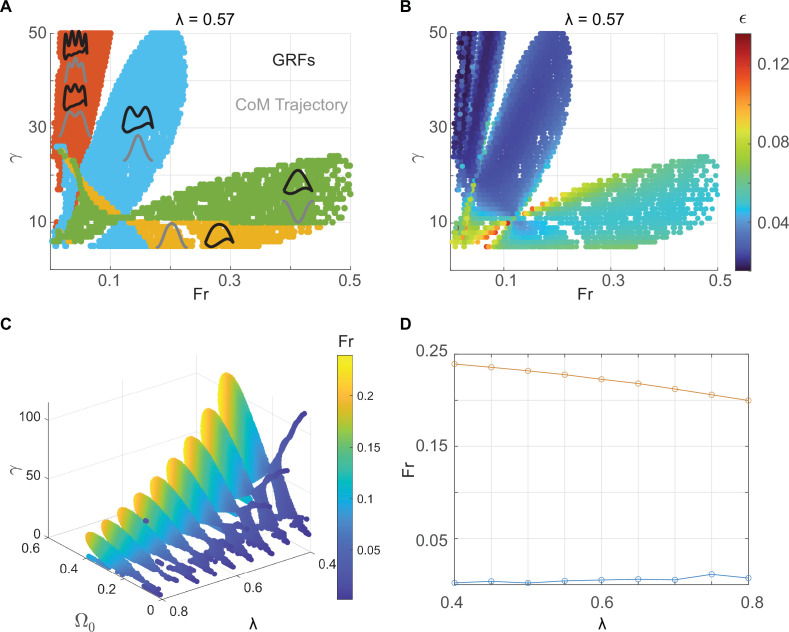
M-shaped walking only occurs only over a limited range of speeds over which it is energetically favoured. (A) Solution space for a fixed value of dimensionless step length, selected according to the best fit to the experimental data for the preferred walking speed of our subject. Four walking modes are shown—three modes from figure 2 and one mode with multiple oscillations. The vGRF is shown in black, and the CoM profile is in grey. (B) The same plot as A, with colours specifying the maximum energy stored in the leg during a cycle, shows the M-shaped GRF is the most energy efficient over the range of speeds for walking. (C) The solution space for M-shaped GRFs for different step lengths. The spring stiffness changes with speed. (D) The M-shaped walking observed in humans is limited to a Fr of 0.25 across step lengths.

The range of speeds, expressed as Froude number, Fr, the square of the dimensionless average velocity (approximately equals ${\Omega_{0}}^{2}$ because the angular speed does not change much during a step), over which limit cycle walking is possible at a given *λ* is shown in figure 3A. Limit cycles with M-shaped vGRF are found only over part of the speed range over which humans typically walk. Consistent with previous work, DSLIP cannot model M-shaped walking at the higher end of walking speeds [2,22,29,33,34]; this limitation of DSLIP will be explored in the next section. Modes with higher oscillations are found only at low speeds (orange region in figure 3A), as going through multiple oscillations takes time, increases stance duration and decreases speed.

The range of speeds for which a single-humped vGRF (inverted gaits) was observed is more extensive than the M-shaped vGRF. At low speeds, both the M-shaped vGRF and the inverted force profiles are possible using different γ values. However, only the inverted force profile is possible at high Froude numbers. Part of this regime (green area) corresponds to grounded running. Consistent with grounded running observed in humans and other bipeds [1,27,30,31], the spring constant decreases as the gait transitions from normal walking to grounded running.

#### 2.1.3. Normal walking gaits with ‘M’-shaped vertical ground reaction force are preferred because they are energetically efficient

Do humans choose M-shaped GRFs during walking (despite other modes being accessible) because it is energetically favourable? Although DSLIP itself is a conservative model, the spring compression modelled by DSLIP will require work proportional to the energy stored in the SLIP spring. The maximum spring energy stored is a proxy for the energy cost of transport during the given walking step. The maximal stored energy is given by


(2.1) $$\begin{array}{lcr} & \epsilon =\frac{1}{2}\gamma \delta_{\max}^{2}\;. & \end{array}$$

The stored energy for a given walking speed, $\Omega_{0}$, for the normal and inverted gaits can be estimated. For the normal gait, $\delta_{\max}\approx 2/\gamma -\delta_{0}$, while in the inverted gaits, $\delta_{\max}\approx \delta_{0}>1/\gamma$ (figure 1). In the normal gait, the minimum ϵ is achieved by choosing $\delta_{0}\to 1/\gamma \Rightarrow \delta_{\max}\to 1/\gamma$, so that


(2.2) $$\begin{array}{lcr} & \epsilon_{\min\;,\mathrm{normal}}\approx \frac{1}{2\gamma }\;. & \end{array}$$

Since $\delta_{0}>1/\gamma$, in the inverted gaits, ϵ is minimized as $\delta_{0}\to 1/\gamma$ as well. Note that for the normal gait, 1/γ is the largest value of $\delta_{0}$, while for the inverted gait, it is the lowest.


(2.3) $$\begin{array}{lcr} & \epsilon_{\min\;,\mathrm{inverted}}=\frac{1}{2}\gamma \delta_{0}^{2}=\frac{1}{2\gamma }\;. & \end{array}$$

For a given speed, the expression for the minimum stored energy is the same for both gaits and is inversely proportional to γ. Therefore, the gait with higher γ—the normal gait (figure 3A)—is preferred. The same can be inferred intuitively: the take-off angle, $\theta_{\mathrm{off}}$, does not change much between different walking trajectories. Thus, the time, $\theta_{\mathrm{off}}/\Omega_{0}$, that a leg is on the ground stays approximately the same as long as the walking speed is the same. However, at this time, during normal walking, the radial coordinate must oscillate once for normal walking and only undergoes half an oscillation for grounded running. Since oscillation frequency goes as the square root of stiffness, γ, the normal walking gait must have a larger stiffness.

To quantitatively test this idea, we evaluated ϵ throughout the space where we have limit cycle solutions and ϵ was smaller for the normal gait compared with the inverted gaits (figure 3B) for the same speed. Therefore, M-shaped vGRFs are preferable to grounded running because they minimize energy. Gaits with multiple oscillations are even more efficient and should be preferred, but these gaits have a maximum attainable speed; the higher the number of oscillations, the smaller this speed bound. Within the preferred speed range of human walking, higher oscillatory modes are not available (or have very large stiffness), making the normal walking gait the most energy-efficient gait.

**Figure 4.**
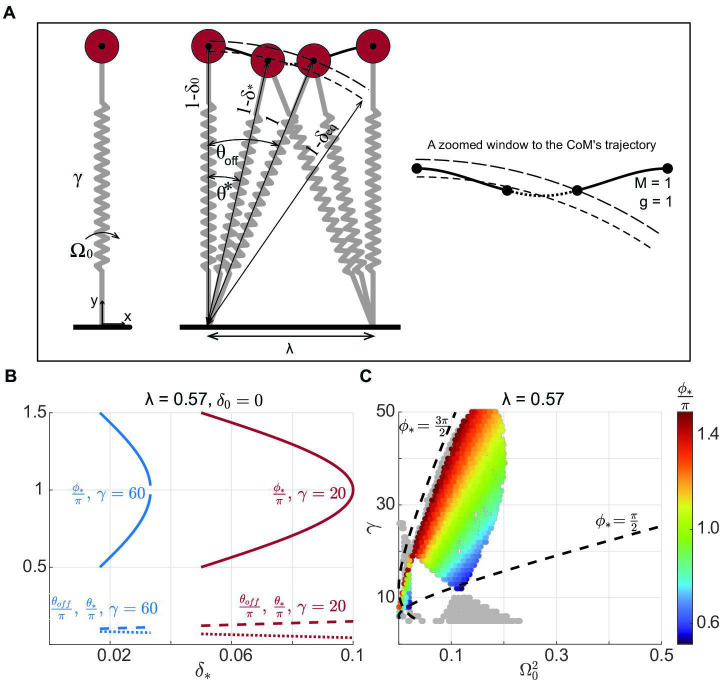
Synchronization between radial and angular motion divides the gait space into regions in which different modes are expected. (A) An example simulation to illustrate synchronization between radial and angular oscillation (middle panel, zoomed version on the right). At a given step length and leg contraction at mid-stance, any γ and $\Omega_{0}$, only solutions that have synchronized radial and angular motion can become a limit cycle. During the time it takes to travel from mid-stance to the transition between single- and double-stance phases—denoted by the starred variables—the angular coordinate must go from mid-stance to $\theta_{⋆}$. The radial coordinate will go from its position between the natural length and equilibrium length at mid-stance to a position slightly below it. This corresponds to a change in the value of ϕ from 0 at mid-stance to $\frac{\pi }{2}<\phi_{⋆}<\frac{3\pi }{2}$ at the transition. (B) Two examples based on analytical results show that while $\phi_{⋆}$ approximately accesses the entire range defined for normal walking, $\theta_{off}$ and $\theta_{⋆}$ slightly increases and decreases, respectively. (C) In the figure, only solutions with $\frac{\pi }{2}<\phi_{⋆}<\frac{3\pi }{2}$ are shown by colour bar; the others are grey. Analytical constraints from synchronization are shown by the dashed line, which is close to the lower bound on speed. However, there is no limit on the upper bound. Note that at high γ, there are no limit cycle solutions close to $\phi_{⋆}=\frac{\pi }{2}$.

The analysis above ignores the energy used to propel the swing leg; an approximate assessment of the energetics of the swing phase shows that normal gait will be preferred. It has been previously proposed that the swing energy is $\propto \nu^{4}$, where $\nu =1/(\tau_{s})$ is the angular frequency of the swing leg, and $\tau_{s}$ is the dimensionless time for the single stance/swing phase [15]. For a given angular speed, the energy will diminish steeply with $\theta_{⋆}\propto \tau_{s}$, or


(2.4) $$\begin{array}{lcr} & \epsilon_{swing}\propto \frac{1}{\theta_{⋆}^{4}}\;, & \end{array}$$

where $\theta_{⋆}$ is the angular coordinate at the transition from the first single stance to the double stance. For geometrical reasons, just like $\theta_{\mathrm{off}}$, $\theta_{⋆}$ does not vary much between different gaits, but it does increase slightly (figure 4B) as one decreases $\delta_{⋆}$. Since an increase in γ decreases $\delta_{⋆}$, gaits with higher γ are preferred.

To investigate the range of speed allowed using the M-shaped GRF pattern, we found the limit cycles for the range of relative step lengths (λ) in our experimental data. The region allowed for M-shaped (normal human walking; figure 3C) shows that as λ decreases, the lowest value of γ allowed increases. The maximum and minimum Froude numbers (Fr) (figure 3D) show that DSLIP is a good model at lower speeds but is limited at higher speeds across λ values. Compared with the previous study [26], which assessed the range of Fr numbers allowed using constraints on the single stance, i.e. without any requirement for limit cycles [26], the allowed speed is only altered at the higher end. Essentially, DSLIP is an adequate model for walking at slow speeds, whether one considers just the synchronization of radial and horizontal motions during the single stance or the full gait cycle. In contrast, the range of speed at the high end dramatically decreases when the double-stance phase is included, a topic discussed at length in the next section.

### 2.2. Constraints from synchronization of radial and angular motion in single stance and velocity redirection in double stance limit double spring-loaded inverted pendulum’s normal walking speed

#### 2.2.1. Synchronization between radial and angular motion during the single stance describes the lower limits of speed possible with M-shaped ground reaction force

The mechanical constraints that limit the range of speeds for M-shaped walking are not understood. To better understand these constraints, we sought an analytical approximation of the DSLIP model. The analytical approximation has two parts that correspond to single- and double-stance phases, respectively (see electronic supplementary material, appendix C for details). First, during the single-stance phase, we assume that the angular and radial motions are decoupled. When there is no angular motion, and θ≈0, the equation of radial motion can be written as


(2.5) $$\ddot{\delta }=-\gamma \left(\delta -\frac{1}{\gamma }\right)\Rightarrow \delta =\frac{1}{\gamma }+\left(\delta_{0}-\frac{1}{\gamma }\right)\cos(\omega t),\text{ where }\omega \equiv \sqrt{\gamma }.$$

In other words, δ simply oscillates around its equilibrium value, 1/γ. Furthermore, under the approximation that angular speed is constant, we have


(2.6) $$\begin{array}{lcr} & \theta =\Omega_{0}t. & \end{array}$$

The oscillation phase of the radial motion can be defined as


(2.7) $$\begin{array}{lcr} & \phi \equiv \omega t. & \end{array}$$

If $\phi_{⋆}$ and $t_{⋆}$ denote the oscillatory phase and time when the single stance transitions to the double stance, at this same time, the angular motion must traverse up to the transition angle, $\theta_{⋆}$ (figure 4A)


(2.8) $$\begin{array}{lcr} & t_{⋆}=\frac{\phi_{⋆}}{\omega }=\frac{\theta_{⋆}}{\Omega_{0}}. & \end{array}$$

In other words, γ and $\Omega_{0}$ are related as


(2.9) $$\begin{array}{lcr} & \Omega_{0}=\left(\frac{\theta_{⋆}}{\phi_{⋆}}\right)\sqrt{\gamma }. & \end{array}$$

This equation implies that as speed ($\Omega_{0}$) increases, the leg must oscillate faster in the radial direction to keep up, leading to a greater stiffness (γ). The relationship between ($\Omega_{0}$) and (γ) is more complex as $\theta_{⋆}$ and $\phi_{⋆}$ are not constants but rather given by (see electronic supplementary material, appendix C)


(2.10) $$\sin\theta_{⋆}=\frac{\lambda^{2}+{\left(1-\delta_{⋆}\right)}^{2}-1}{2\left(1-\delta_{⋆}\right)\lambda },\text{ and }\cos\phi_{⋆}=-\left(\frac{\gamma \delta_{⋆}-1}{1-\gamma \delta_{0}}\right).$$

Briefly, the $\theta_{⋆}$ results from the transition geometry (figure 4A), and $\phi_{⋆}$ from equation (2.5). Since $\delta_{⋆}$ is typically small and ranges between $1/\gamma <\delta_{⋆}<2/\gamma \ll 1$, $\theta_{⋆}$ does not change much; there is a small increase with decreasing $\delta_{⋆}$ (figure 4B). Assuming γ≫1, we have


(2.11) $$\frac{\lambda }{2}\left(1-\frac{2}{\gamma \lambda^{2}}\right)≳\sin\theta_{⋆}≳\frac{\lambda }{2}\left(1-\frac{4}{\gamma \lambda^{2}}\right)\;.$$

As γ increases, $\delta_{⋆}$ becomes smaller, and accordingly $\theta_{⋆}$ increases towards $\sin^{-1}\left(\lambda /2\right)$.

In contrast to the small $\theta_{⋆}$ range, $\phi_{⋆}$ changes considerably (figure 4B). When $\cos\;\phi_{⋆}$ is negative (we will justify this in the next subsection), $\phi_{⋆}$ can, *a priori*, take any value in the range


(2.12) $$\begin{array}{lcr} & \frac{\pi }{2}<\phi_{⋆}<\frac{3\pi }{2} & \end{array}$$

for a single radial oscillation of the CoM. Moreover, (2.10) implies that as $\delta_{⋆}$ varies, we have two branches of $\phi_{⋆}(\delta_{⋆})$: a branch along which $\delta_{⋆}$ varies between 1/γ and $2/\gamma -\delta_{0}$ and $\phi_{⋆}$ varies between π/2 and π (see figure 4B), and another where $\phi_{⋆}$ goes from π to 3π/2 as $\delta_{⋆}$ varies between $2/\gamma -\delta_{0}$ and 1/γ.

We can estimate the speed bounds based on the analytical equations above. It is clear from (2.9) that $\Omega_{0}$ decreases if $\phi_{⋆}$ increases and $\theta_{⋆}$ decreases; however, the effect of $\theta_{⋆}$ change is much larger. Thus, approximately the lower bound on speed is attained at $\delta_{⋆}\to 1/\gamma$ and $\phi_{⋆}\to 3\pi /2$, following the upper branch and yielding


(2.13) $$\begin{array}{lcr} & \Omega_{0}≳\left(\frac{2}{3\pi }\;\sin^{-1}\left[\frac{\lambda }{2}\left(1-\frac{2}{\gamma \lambda^{2}}\right)\right]\right)\sqrt{\gamma }. & \end{array}$$

In a similar way, the upper bound is attained as $\delta_{⋆}\to 1/\gamma$ and $\phi_{⋆}\to \pi /2$


(2.14) $$\begin{array}{lcr} & \Omega_{0}≲\left(\frac{2}{\pi }\;\sin^{-1}\left[\frac{\lambda }{2}\left(1-\frac{2}{\gamma \lambda^{2}}\right)\right]\right)\sqrt{\gamma }. & \end{array}$$

The upper and lower bounds resulting from this synchronization are plotted in figure 4C. The analytical lower bound derived above matches the simulation results well, implying that the analytical approximation captures the mechanics well. However, the analytical upper bound does not match the bounds obtained through simulation. This mismatch occurs because, except for low γ, the allowed $\phi_{⋆}$ does not reach π/2; the allowed $\phi_{⋆}$ deviates further from π/2 as γ increases. Single-stance mechanics do not constrain the speed for normal walking; instead, as we will see next, constraints from double support limit $\phi_{⋆}$. This result explains why a previous study that considered only the single-support phase came to the conclusion that DSLIP can function as a model for walking even at high speeds [26].

#### 2.2.2. Limits of double spring-loaded inverted pendulum on speed result from a combination of synchronization and the requirement to redirect vertical velocity component during the double-stance phase

**Figure 5.**
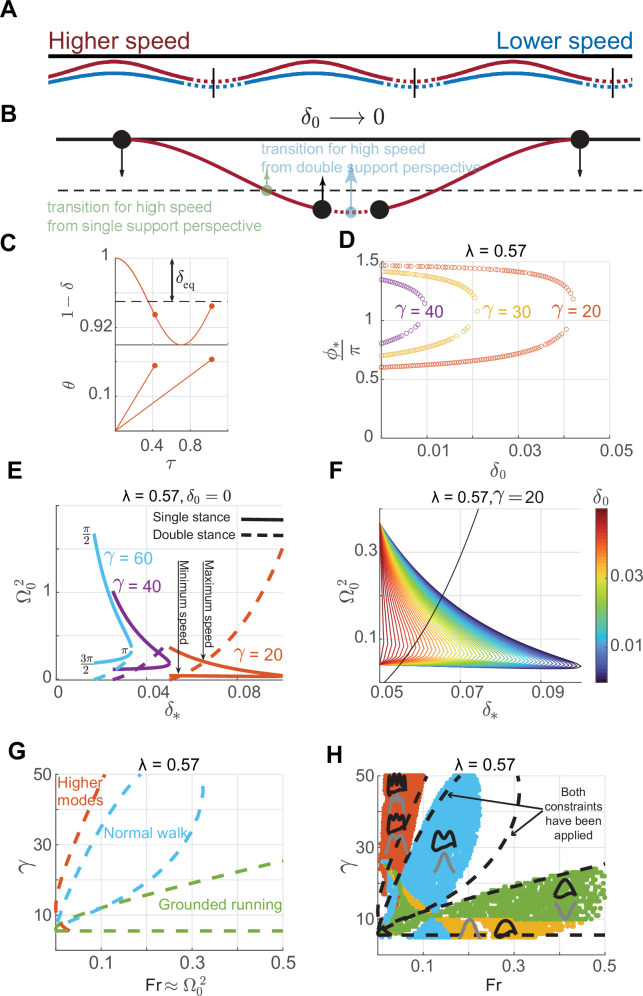
M-shaped walking is limited to low speeds because of a combination of synchronization and velocity redirection constraints. (A) Two CoM trajectories illustrate the single-stance (solid lines) and the double-stance (dotted lines) phases. The double-stance phase gets shorter with increasing speeds. The vertical black lines specify the mid-step. (B) The conditions preferred from the single-stance synchronization viewpoint (green solid) for high speeds are different from those that will produce the largest redirection forces (cyan). The actual transition point is somewhere between the two extremes. This compromise means that the speeds allowed for M-shaped GRF walking are limited. (C) The figure shows the approximate evolution of δ and θ during the single-stance phase. There are only two solutions once γ, $\delta_{0}$, λ are fixed. (D) The variation of $\phi_{⋆}$ versus $\delta_{0}$ for a given step length for the normal walking gait. By increasing γ, generally the speed increases, and for a given gamma, as the speed increases, $\phi_{⋆}$ and $\delta_{0}$ values get closer to π and zero, respectively. (E) A graphical representation of how single- (solid line) and double-stance (dashed line) constraints affect the range of possible speeds. Here, $\delta_{0}=0$. The highest speed possible (intersection) is much smaller than the highest speed from just single-stance considerations (obtained at $\delta_{⋆}=\frac{1}{\gamma }$). The difference becomes more with higher stiffness until at the highest stiffness (light blue, γ = 60), there is no solution (no intersection point). (F) All solutions for a fixed step length and stiffness. Note that the double-stance constraint is independent of $\delta_{0}$. (G) The region of different gait patterns that is estimated by our analytical approximation. The boundaries for normal walking become highly constrained. The other boundaries—for grounded running and higher modes—are a result of single-stance constraint alone. (H) Analytical boundaries of walking solutions from G overlayed on the numerical solution for comparison.

The vertical CoM velocity, which is pointed downward at the beginning of the double-support phase, must be redirected upward at the end of the double-support phase [2]; the required redirection increases with speed. As speed increases, this redirection becomes more difficult because the double-support phase becomes shorter, and the required change in velocity is larger (figure 5A): as speed increases, γ increases as well, and so does the equilibrium height (1−1/γ). Moreover, as the radial motion of the CoM is approximately oscillating with an amplitude less than 1/γ, the CoM trajectory is closer to the natural leg length at higher speeds (figure 5A), r≲1−2/γ. Consequently, the transition geometry dictates that the transition occurs closer to the mid-step at higher speeds. This change, in conjunction with increased horizontal speed, implies that less time is spent in the double-support phase. At the same time, as the vertical component of velocity increases with the overall speed increase, a larger change in speed is required at the transition. A larger speed change in a shorter time necessitates a larger acceleration. A back-of-the-envelope calculation is instructive: the double-support phase duration, $t_{d}∼\delta \theta /\Omega_{0}$, keeps shrinking as speed increases, while the required change of vertical velocity necessary during the double-support phase increases, $\delta v∼2\Omega_{0}\sin\;\theta_{⋆}$. Thus, the average upward acceleration that one needs, $∼\delta v/t_{d}\approx \Omega_{0}^{2}\sin\;\theta_{⋆}/\delta \theta$, increases with speed. The increased acceleration cannot be produced because the force that can be generated during normal walking is bounded and is equal to the weight (this is the maximum downward force one can have, and in the simple harmonic approximation, it equals the maximum upward force). Moreover, we will show that in contrast to the synchronization during the single-support phase, where the highest speeds are attained when $\phi_{⋆}$ is close to π/2, a successful velocity redirection is more likely when $\phi_{⋆}$ is close to π. This mismatch between the conditions that support the highest velocity in the single- and double-support phases severely limits the speed bound for M-shaped GRF (figure 5B). This mismatch is illustrated in figure 5B and only exists for high speeds. The above arguments are qualitative. Below, we provide an approximation and an analytic condition determining the maximum speed bound for the normal walking gait.

To estimate the speed bound, we will first derive an approximate analytical solution for the limit cycle and then use this analytical solution to estimate the speed bound. The horizontal speed and vertical acceleration are approximately constant during the double-stance phase and equal to their value at the transition between single- and double-stance phases: $v_{x}\approx v_{x⋆}$ and $a_{y}\approx a_{y⋆}$. In particular, this constancy implies that at the transition, the vertical acceleration must be upward to make velocity redirection possible, or $a_{y⋆}>0$. For an approximately simple harmonic radial oscillation, this occurs during the phase, $\frac{\pi }{2}≲\phi_{⋆}≲\frac{3\pi }{2}$, thereby justifying the assumption (2.12) we made earlier. Using these approximations, and the fact that in the time the leg has to travel horizontally to the mid-step from the transition point, the upward force must be sufficient to bring the downward velocity at transition to zero at mid-step, we can derive the relationship $\Omega_{0}(\delta_{0},\delta_{⋆},\gamma ,\lambda )$ (see electronic supplementary material, appendix D for the derivation) as


(2.15) $$\begin{array}{lcr} & \Omega_{0}^{2}=\frac{\left(\gamma \delta_{⋆}\cos\;\theta_{⋆}-1)[\lambda /2-(1-\delta_{⋆})\;\sin\;\theta_{⋆}\right]}{(1-\delta_{⋆}{)}^{2}\sin\;\theta_{⋆}\cos\;\theta_{⋆}}\equiv G_{D}(\delta_{0},\delta_{⋆},\gamma ,\lambda ). & \end{array}$$

This nonlinear function determining $\Omega_{0}$ as a function of $\delta_{0},\delta_{⋆},\gamma ,\lambda$ describes the speed based on the double-stance constraint. Because $\delta_{⋆}\ll 1$ and $\theta_{⋆}$ approximately remain a constant, the first term (the net upward force) in the numerator is the most important for determining speed, and this will be important later.

The synchronization relation obtained from the single-stance phase is also a function of $\delta_{0},\delta_{⋆},\gamma ,\lambda$


(2.16) $$\begin{array}{lcr} & \Omega_{0}^{2}={\left(\frac{\theta_{⋆}}{\phi_{⋆}}\right)}^{2}\gamma \equiv G_{S}(\delta_{0},\delta_{⋆},\gamma ,\lambda ). & \end{array}$$

Thus, in order to have a synchronized limit cycle, the four parameters, $\delta_{0},\delta_{⋆},\gamma ,\lambda$, must be related


(2.17) $$\begin{array}{lcr} & G_{D}(\delta_{0},\delta_{⋆},\gamma ,\lambda )=G_{S}(\delta_{0},\delta_{⋆},\gamma ,\lambda ), & \end{array}$$

leaving only three independent parameters, $\delta_{0},\gamma ,\lambda$. For a given $\delta_{0}$ and γ, inverting the cosine function in (2.10) while obtaining $\phi_{⋆}$ results in two branches, referred to here as $\phi_{u}(\delta_{⋆})\in \left(\pi ,3\pi /2\right)$ and $\phi_{l}(\delta_{⋆})\in \left(\pi /2,\pi \right)$ (figure 5D). Accordingly, for a given λ,γ and $\delta_{0}$, the upper branch, $\phi_{u}$, leads to a branch with lower speeds from the single-stance synchronization condition (2.16)


(2.18) $$\begin{array}{lcr} & \Omega_{0}^{2}={\left[\frac{\theta_{⋆}(\delta_{⋆})}{\phi_{u}(\delta_{⋆})}\right]}^{2}\gamma \equiv G_{l}(\delta_{0},\delta_{⋆},\gamma ,\lambda ), & \end{array}$$

while the lower branch leads to a branch with higher speeds


(2.19) $$\begin{array}{lcr} & \Omega_{0}^{2}={\left[\frac{\theta_{⋆}(\delta_{⋆})}{\phi_{l}(\delta_{⋆})}\right]}^{2}\gamma \equiv G_{u}(\delta_{0},\delta_{⋆},\gamma ,\lambda ), & \end{array}$$

So, if the three parameters, $\delta_{0},\gamma ,\lambda$, are fixed, there are only two possible values of $\Omega_{0}$ resulting from two values of $\phi_{⋆}$ and $\delta_{⋆}$ corresponding to two branches of solution (figure 5C); from a different perspective, relating single and double stance dramatically shrinks the solution space from the entire range between π/2 and 3π/2 for allowed $\phi_{⋆}$ to just two values of $\phi_{⋆}$ (figure 5D).

#### 2.2.3. Satisfying both single- and double-stance constraints simultaneously is difficult at high speeds and curtails speeds at which walking is possible

Normal walking must satisfy both the single- and double-stance requirements (2.17). The maximum speed occurs at different $\delta_{⋆}$ and $\phi_{⋆}$ values for the single and double stance: synchronization during single stance (2.16) suggests that a speed maximum is reached as $\delta_{⋆}\to 1/\gamma$ and $\phi_{⋆}\to \pi /2$ (figure 5E). However, synchronization during double stance does not allow $\delta_{⋆}\to 1/\gamma$ and $\phi_{⋆}\to \pi /2$: as $\delta_{⋆}\to 1/\gamma$, the upward force (the first term within the parentheses in the numerator of (2.15)) becomes negative and is disallowed (figure 4C). Thus, it is not possible for $\phi_{⋆}$ to attain π/2 (figure 5E). This inability of $\phi_{⋆}$ to reach π/2 is also reflected in the simulation results in figure 4C and becomes worse as γ increases (figures 4C and 5D). The maximum upward force in the double-stance phase occurs at the largest compression possible, $\delta_{⋆}\approx 2/\gamma$, when $\phi_{⋆}\approx \pi$. In calculating the force, $\delta_{⋆}$ is multiplied by γ≫1, and, thus, the effect of $\delta_{⋆}$ in $G_{D}$ is dominated by the force term. The maximum speed possible is a compromise between the considerations from single and double stance, and the largest speed occurs at a value of $\phi_{⋆}$ between π/2 (where the maximum speed from single-stance condition occurs) and π (where the maximum speed from double-stance condition occurs).

By inspection of (2.10), it is also clear that for a given $\delta_{⋆}$, $\phi_{⋆}$ is smallest if $\delta_{0}=0$. Thus, the maximum speed is approximately attained at a $\delta_{⋆}$ that satisfies both (2.16) and (2.15) for $\delta_{0}=0$. Or,


(2.20) $$\begin{array}{lcr} & {\left(\frac{\theta_{⋆}}{\phi_{⋆}}\right)}^{2}\gamma =\frac{\left(\gamma \delta_{⋆}\cos\;\theta_{⋆}-1)[\lambda /2-(1-\delta_{⋆})\;\sin\;\theta_{⋆}\right]}{(1-\delta_{⋆}{)}^{2}\sin\;\theta_{⋆}\cos\;\theta_{⋆}}, & \end{array}$$

where $\cos\;\phi_{⋆}=-\left(\gamma \delta_{⋆}-1\right)$, and $\theta_{⋆}$ is given by (2.10). Equation (2.20) can be solved to obtain $\delta_{⋆}$ as a function of λ and γ. Graphically, the solution is given as the intersection between curves depicting equations (2.15) and (2.19) or (2.18) (figure 5E). The constraining function, $G_{D}$, from the double-support does not depend on $\phi_{⋆}$ and, therefore, has no branches. It is a monotonically increasing function of $\delta_{⋆}$ that can intersect both the lower and the higher branches, $G_{l}(\delta_{⋆})$ and $G_{u}(\delta_{⋆})$, leading to two possible solutions. The maximum speed is given by the intersection of these two constraints that occur between $\phi_{⋆}$ of π/2 and π, and is, therefore, lower than the speed possible if we only consider single-stance synchronization. This decrease is exacerbated as γ increases (figure 5E). For a given λ and large enough γ’s, there are no solutions at all, consistent with our numerical findings (figure 5E; γ=60). The lower bound is also attained when $\delta_{0}\to 0$, since this decreases $\cos\;\phi_{⋆}$, allowing $\phi_{⋆}$ to get close to 3π/2 (figure 5E). The lower bound is reached when $\delta_{⋆}$ is close to 1/γ, but as argued before, the upper bound $\delta_{⋆}$ ends up at a compromise value between 1/γ and 2/γ. The effect of the double-stance constraint on the lower speed bound is much less (figure 5E).

Essentially, the same analysis can be performed for non-zero $\delta_{0}$ with two limit cycles possible for a given value of $\delta_{0}$. More generally, the double-valued nature of $\phi_{⋆}(\delta_{⋆})$ leads to a double-valued $\delta_{⋆}(\delta_{0})$ function (figure 5F), resulting in a family of curves—one for each $\delta_{0}$.

The overall results are summarized in figure 5G,H. The single-stance constraint alone divides the gait space into contiguous regions with different oscillatory gaits (figure 4C). Addition of the double-stance constraint limits the region allowed (figure 5G). The results from the analytical approximation of DSLIP and the actual simulations are overlayed in figure 5H. The range of speeds predicted from the analytical consideration (see electronic supplementary material, appendix D for more details) matches the simulation results closely. The correspondence is particularly close for low speeds. The small discrepancy at the higher speed is probably a result of the oversimplification of the dynamics of the double-stance phase. However, the critical result is that it is the differing constraints from synchronization in the single- and double-stance phases that limit the range of speed over which M-shaped walking is possible. Two other features of the gait space are described in detail in electronic supplementary material, appendix F. First, there is a lower bound on γ. Second, the analysis in this section extends to gaits with multiple oscillations.

### 2.3. Double spring-loaded inverted pendulum is an adequate model for human walking only for a narrow range of speeds

**Figure 6.**
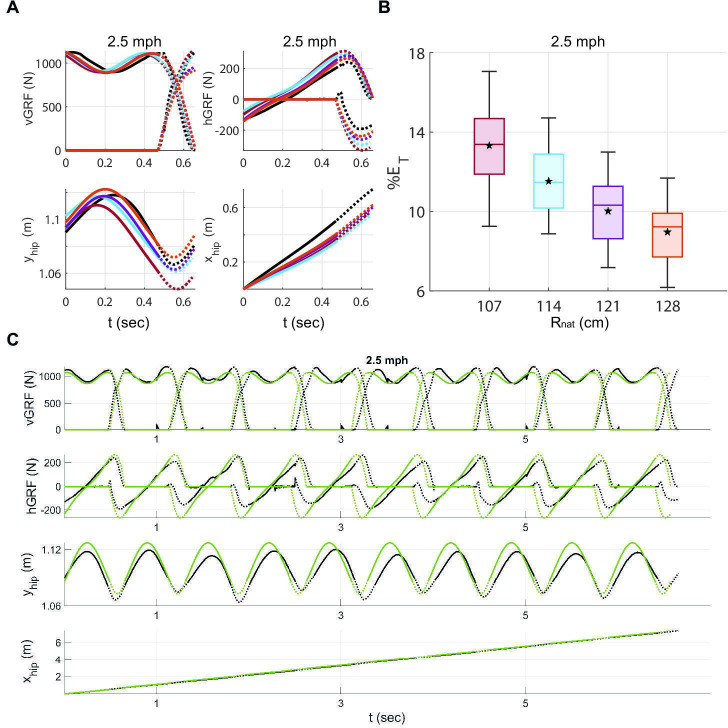
DSLIP is an excellent model for human walking over a narrow range of speeds. (A) Since the hip marker may not be exactly at the CoM, we fit the experimental data (black lines) to a range of heights both smaller and larger than the hip height (coloured lines; see electronic supplementary material, A.1). Solid lines and dotted lines represent single- and double-stance phases, respectively. (B) The total error for each leg length shows that 128 cm has the lowest error. The error is the sum of errors related to the vGRF, hGRF, height and horizontal displacement of the CoM. (C) The optimized limit cycle based on $R_{nat}=128\text{cm}$ (green lines) fits well into 10 walking steps. The total error in time is negligible.

To evaluate whether the interactions between the walker and the substrate can be quantitatively described with a spring-mass model, we next evaluated how close DSLIP came to describing the kinematics and GRFs during walking. To this end, we fit DSLIP to human walking data. Using an instrumented treadmill, we collected data for four walking speeds—2.0, 2.5, 3.0 and 3.5 miles per hour (mph; see electronic supplementary material, S4.2). Following previous work [26], we fit the GRF and CoM kinematics in real-world units or dimensional units and individual steps instead of the average data. As choosing the height of the hip marker is a good approximation for the movement of the CoM in time but not the exact CoM location, we began by determining the optimal $R_{nat}$ for 2.5 mph, which was the preferred walking speed for the subject (figure 6A).

To this end, we first fit a non-periodic trajectory, i.e. the fits were not constrained to be limit cycles, to each walking step separately, to allow more flexibility and independent assessment of the best fit over 40 steps, thereby increasing statistical power (see electronic supplementary material, A.1). In obtaining $R_{nat}$, we used four values of $R_{nat}$. The vertical GRF was well fit at all selected values of $R_{nat}$, as was the height of the CoM. The highest value of $R_{nat}$, 128 cm, was the best fit to the horizontal GRF (figure 6A,B), yielded the lowest overall error and was selected for limit cycle fits.

After fixing $R_{nat}$, there remained only three free parameters that determine a limit cycle; two of them—the average step length and speed—were fixed by constraining them to match the experimentally observed step length and step time. The remaining parameter is selected as the average minimum vGRF over the single-stance phase, which can be directly calculated from the data as well.

One example of the limit cycle fit is shown in figure 6C. A single limit cycle closely describes the entire sequence of steps rather than the average step as is typically done; as a consequence, the limit cycle fits some steps better than others. As an example, the fourth step, which is slower than the optimized limit cycle, does not fit well, but this delay is corrected by faster steps later in the sequence (figure 6C). Overall, a single limit cycle optimized to fit the entire sequence of steps fits the data well and implies that DSLIP is an excellent model for walking at the preferred speed.

**Figure 7.**
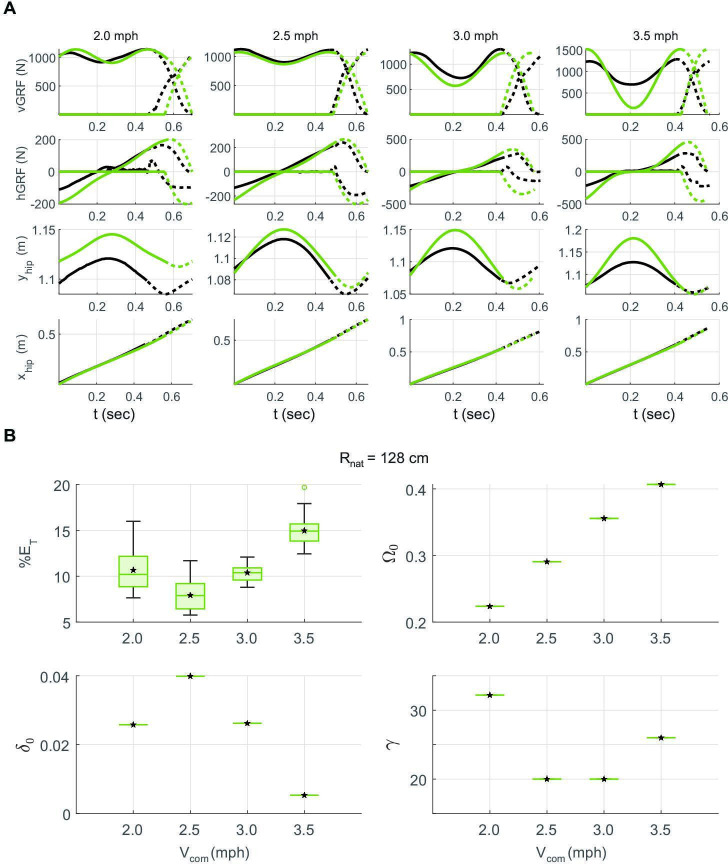
DSLIP fits for both lower and higher than the preferred speed are worse, but for distinct reasons. (A) Example fits (green lines) and data (black lines). Solid lines and dotted lines represent single- and double-stance phases, respectively. The model and subject have the same step length and speed in all fits. We optimized limit cycles based on the values of vGRF at the mid-stance, which can be considered the only free parameter left. The best fit belongs to the preferred speed (2.5 mph), and the highest speed (3.5 mph) has the worst prediction. (B) The total errors including GRFs and CoM kinematics along with the parameters of the optimized limit cycles for different walking speeds.

Typical single-step fits, one for each of the four speeds, are shown in figure 7A. Walking at 2.5 mph is best modelled by DSLIP; at this speed, the optimized limit cycle tracks important dynamical features such as the step length, speed, vGRF and the single-stance time (electronic supplementary material, figure S1D). The model still produces reasonable fits at both 2.0 and 3.0 mph, but the fits deteriorate at these speeds. At 2.0 mph, the best-fit model has a longer single-stance time; the fitted vGRF oscillates somewhat more than the empirical data. The nature of the deviation is different at 3.0 mph where the model has a lower minimum in vGRF compared with the subject and much larger vertical CoM oscillations. The model completely fails at 3.5 mph as the minimum in the vGRF is close to zero. The average of total errors from GRFs and CoM kinematics, along with parameters of optimized limit cycles, is shown in figure 7B. The total error validates our qualitative observations above, showing median errors of less than 10% at 2.0, 2.5 and 3.0 mph and larger for fits at 3.5 mph.

**Figure 8.**
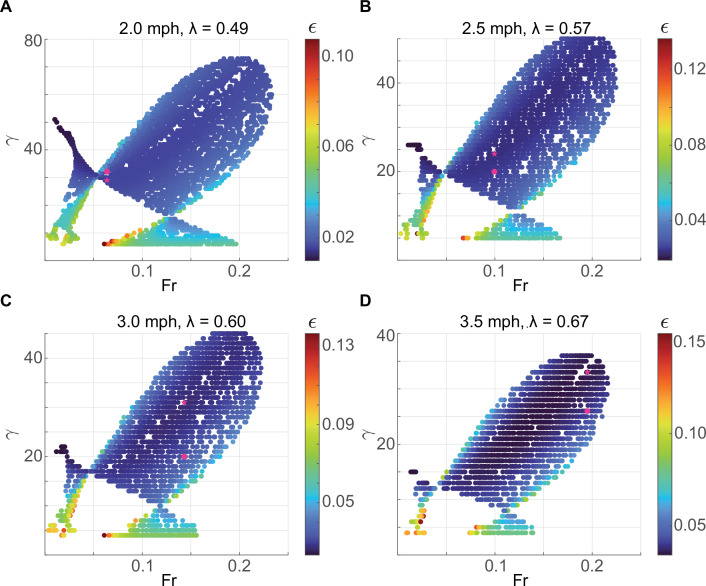
The range of spring constants where a limit cycle is possible probably makes it difficult to obtain good fits for human walking. (A–D) belong to the 2.0, 2.5, 3.0 and 3.5 mph walking speeds of the subject, respectively. The pink circles show the optimized limit cycles based on our method, and the pink stars show the limit cycles with the minimum energy at the same speed. At both 2 and 3.5 mph, the optimization solutions are close to the solution boundary.

Surprisingly, the best-fit spring constant is higher for 2.0 mph (figure 8A); this finding provides one important clue regarding why DSLIP works as a great model for walking at 2.5 mph and not other speeds. The higher spring constant is unexpected because most previous work has shown that the spring constant decreases as the speed decreases [35,36]. Indeed, the spring constant for the single-stance phase, as directly inferred from the force–length curve, decreases with speed (electronic supplementary material, figure S2). At the step length used by our subject to walk at 2.0 mph, there are no limit cycle solutions for this spring constant (figure 8B), and therefore, the spring constant for the best-fit limit cycle is artificially higher. Previous work [37] suggested that at low speeds, it becomes increasingly important to model tangential forces. Their introduction may allow one to walk with lower values of γ in this low velocity regime and provide a more accurate description of the dynamics. The force–length relationship (electronic supplementary material, figure S2) also shows that at 2.5 mph, the spring constant during single- and double-stance phases is similar, which explains why a DSLIP model that uses a single spring constant is a quantitative model for human walking at that speed. At higher speeds, the spring constants that describe single- and double-stance phases become very different, and this difference makes it difficult for the DSLIP model to describe the data. The fits at 3.0 mph have a stiffness that is intermediate between single- and double-stance stiffnesses. As a result, the model fit has a smaller γ than suggested from the force–length measurements in the single-stance phase. With this smaller stiffness, generation of the observed fluctuations in vGRF required a much larger change in the CoM height. In sum, DSLIP seems to function as a quantitative model around the preferred walking speed. At lower speeds, the range of spring constants that can lead to limit cycles shrinks. At higher speeds, the spring constants that describe single- and double-stance phases are different, making it difficult for DSLIP to model.

## 3. Discussion

### 3.1. A compliant leg is necessary for modelling many features of locomotion

A model with non-compliant legs—IP—continues to persist as a model for walking. The IP model has been successful in explaining the energetics of walking [13–15]. The inability of the IP model to describe forces is considered a surmountable limitation: by relaxing the impulsive nature of work in the IP model, the model can recover the M-shaped GRF observed during walking. However, we show here that a compliant leg is necessary for modelling many essential features of locomotion, including gait choice and speed-dependent gait transitions. First, regarding gait choice, by providing a means to relate leg stiffness that controls the amplitude and period of the vertical oscillation to the angular speed of stance progression, leg compliance provides an analytical framework rooted in mechanics for analysing which gaits will be observed. In analysing gait choice, leg compliance is necessary as many of the gaits are not available in the stiff-legged model. We also show here that the energetics of a compliant leg are necessary for understanding why a particular gait, defined by GRF and kinematics, is observed in a given step. Perhaps more importantly, leg compliance is necessary for understanding speed-dependent gait transition, as the reasons for gait transition in a stiff-limbed walker are completely different from those with compliant legs. It is clear from the analysis performed in this study that the non-holonomic dynamics, which result in different optimal transition points for single- and double-support phases, limit the range of speeds over which humans can walk.

### 3.2. M-shaped ground reaction forces are prevalent because they are energetically efficient

An unexplained characteristic of human walking is that humans walk with an M-shaped GRF profile. The M-shaped GRF is observed in other walkers, including both bipeds and quadrupeds [38–40]. At the speeds at which humans walk, other modes of walking, such as grounded running, are possible. However, the M-shaped profile is energetically favoured. We have shown that the normal walking gait has a stiffer leg as compared with grounded running, which is preferred because a stiff leg results in smaller vertical oscillations and therefore ultimately less work. This same logic would posit that even higher modes of oscillation with even stiffer legs would be more energy efficient than the normal gait. Accordingly, we observe multi-oscillatory gaits at low speeds (electronic supplementary material, figure S3). These gaits are not available at typical walking speeds. The unavailability of higher oscillatory modes together with the energy efficiency argument selects the single oscillatory normal walking gait with the characteristic ‘M’-shaped vGRF for mammals.

### 3.3. Gait transition occurs because velocity redirection is difficult

An important issue that has received much attention is gait transitions: at what speeds do they happen and why? One approach to this problem is using the IP model. Walking using an IP model is not possible at high speeds because at high speeds—above Fr of 1—the centrifugal force cannot be cancelled by gravitational force. This logic was later modified to take into account the fact that the vertical component of the gravitational force would be lowest near the end of the step [10], which predicts a transition speed of Fr ∼0.5. This argument becomes invalid in compliant legs, which allows part of the centrifugal forces to provide radial acceleration. Moreover, in transitions with double-stance phases, the leg take-off is not a problem as transitions are between double and single stance. Analysis in this study using the DSLIP model comes to a different conclusion for gait transitions. First, even if we take a nuanced approach to walking and impose the condition that walking must have a vGRF minimum at mid-stance, centripetal force does not pose a stringent constraint (figure 4C). Moreover, DSLIP makes it possible to walk with gaits that are not possible using the IP model such as the grounded running gait. In sum, adding compliance to the leg removes the appearance of unphysical negative tension force as a reason for gait transition.

The reason for gait transitions in a compliant walker is completely different, and (to us) highly non-intuitive, and involves three factors: the existence of a vGRF minimum at mid-stance (a defining feature of normal walking), synchronization of horizontal and vertical motions during the single stance and velocity redirection in the double stance. The single-stance synchronization implies that the highest speed at any given spring stiffness occurs when the single- to double-support transition occurs at a different phase (in the oscillatory walking cycle) compared with the phase preferred for the velocity redirection. This tension between single-stance synchronization and double-stance velocity redirection is further accentuated by the requirement of vGRF minimum at mid-stance, which effectively limits the maximal vertical force the legs can exert during double support to affect velocity redirection. It is this tension between the constraints from the single- and double-support phases that results in M-shaped GRFs being impossible as a gait at high walking speeds. There are two options when transitioning from M-shaped walking: transition can be to other walking modes, such as grounded running and inverted walking, or to running with an aerial phase. Thus, analysis using the DSLIP model suggests two different answers to gait transitions: transitions out of M-shaped GRFs occur at low speeds, but transitions from locomotion without an aerial phase to one with an aerial phase can occur at any speed. Both grounded running and aerial running can occur over a large range of speeds.

At what speed aerial running is preferred depends on the individual and species. In humans, transitions can occur from M-shaped walking to aerial running, as is suggested by some. Under certain conditions, there is a small range of speed over which humans walk with a grounded running gait [41,42]. In many birds, grounded running is preferred over a large range of speeds, often exceeding a Fr of 1 [27]. Many non-human primates also prefer grounded running [32]. Fast-running insects and spiders prefer grounded running [43]. To address which gait is preferred, energy estimates for aerial and grounded running at a given speed must be made, which is beyond the scope of this paper.

### 3.4. Limitations of double spring-loaded inverted pendulum and how they might be overcome

DSLIP is a great conceptual model, but with its simplicity comes some limitations. Among them is the fact that DSLIP cannot support M-shaped vGRF walking beyond 0.25, whereas humans can walk with M-shaped vGRF up to a Fr number of 0.45 [44]. There are many mechanisms that might contribute to humans walking at higher Fr numbers. One mechanism is that human legs are not massless, and recoil from the leg swinging forward contributes to velocity redirection [16]. Another mechanism is that the centre of pressure moves forward during stance; this forward movement might increase the range of speeds.

All of these processes can be modelled as additions to the DSLIP model, and aspects of these processes have been explored by others [29,45,46]. Adding features to the model will increase model complexity; the two additions below can be highly beneficial without increasing model complexity. One addition is to use a variable spring stiffness. Both the spring constant and the natural leg length during the single- and double-stance phases are different at high walking speed (electronic supplementary material, figure S2). This difference suggests that changing the stiffness and natural length of the spring during the double-stance phase may be a mechanism for increasing the speed at which M-shaped GRF walking gaits are possible.

Another mechanism is adding an angular spring. As has been noted previously, net forces during walking do not point along the leg but are more vertical [24,26,47]. This limitation can be addressed by adding an angular spring as we have proposed earlier [26,37]. An angular spring produces restorative forces such that there is no angular force at mid-stance. The angular forces increase as the leg moves away from mid-stance. As investigated in [37], such angular forces can provide a much wider range of realistic gaits at low speeds.

## 4. Material and methods

In this section, we briefly describe the model, the essential details related to the empirical data and the numerical techniques to find walking solutions and optimized trajectories. Details are given in the electronic supplementary material.

### 4.1. Walking dynamics of double spring-loaded inverted pendulum

The model is the same as the one introduced by Geyer *et al.* [2]. It has two degrees of freedom that describe the sagittal plan motion of a point mass under gravity and spring forces.

#### 4.1.1. The equations of motion

The model in its full dimension and dimensionless form is shown in figures 1A and 4A, respectively. Figure 1A is a schematic, but figure 4A is based on simulation. The model consists of two massless springy legs hinged with a large mass, *M*, at the hip (CoM). The model does not include any swing phase dynamics, so the single-stance phase is described by just a single spring with the mass at the top. The natural leg length of the springs is denoted by $R_{nat}$. The leg stiffness, $K_{s}$, and the step length, L, are made dimensionless according to the following equations:


(4.1) $$\begin{array}{rl} \gamma & =\frac{K_{s}R_{nat}}{Mg}\\ \lambda & =\frac{L}{R_{nat}}, \end{array}$$

where g is the gravitational acceleration. The dynamics during the single-stance phase evolve according to the following equations represented in the Cartesian form:


(4.2) $$\begin{array}{rl} \ddot{x} & =\frac{\gamma x(1-\sqrt{x^{2}+y^{2}})}{\sqrt{x^{2}+y^{2}}}\\ \ddot{y} & =\frac{\gamma y(1-\sqrt{x^{2}+y^{2}})}{\sqrt{x^{2}+y^{2}}}-1, \end{array}$$

where x and y denote the dimensionless form of horizontal and vertical displacement of the CoM, respectively


(4.3) $$\begin{array}{rl} x & =\frac{X_{com}}{R_{nat}}\\ y & =\frac{Y_{com}}{R_{nat}}. \end{array}$$

Also, we made the time dimensionless by defining


(4.4) $$t^{\prime }=t\sqrt{\frac{g}{R_{nat}}}.$$

Then, the initial conditions are specified by the position and velocity of the CoM at the mid-stance,


(4.5) $$\begin{array}{rl} x_{0} & =0\\ {\dot{x}}_{0} & =(1-\delta_{0})\Omega_{0}\\ y_{0} & =1-\delta_{0}\\ {\dot{y}}_{0} & =-{\dot{\delta }}_{0}, \end{array}$$

where $\delta_{0}$ and $\Omega_{0}$ are the initial dimensionless spring contraction and angular velocity at the mid-stance, respectively. Touch-down occurs at a predefined step length. At this moment, the following algebraic equation is satisfied by the CoM position:


(4.6) $$(\lambda -x{)}^{2}+y^{2}=1.$$

Following touch-down, both the velocity and acceleration of the swing foot become zero, and the governing equations change as follows:


(4.7) $$\begin{array}{rl} \ddot{x} & =\frac{\gamma x(1-\sqrt{x^{2}+y^{2}})}{\sqrt{x^{2}+y^{2}}}-\frac{\gamma (\lambda -x)(1-\sqrt{(\lambda -x{)}^{2}+y^{2}})}{\sqrt{(\lambda -x{)}^{2}+y^{2}}}\\ \ddot{y} & =\frac{\gamma y(1-\sqrt{x^{2}+y^{2}})}{\sqrt{x^{2}+y^{2}}}+\frac{\gamma y(1-\sqrt{(\lambda -x{)}^{2}+y^{2}})}{\sqrt{(\lambda -x{)}^{2}+y^{2}}}-1. \end{array}$$

When the contact force at the trailing leg becomes zero, the leg reaches its natural length and leaves the ground. This moment is called toe-off and is defined by


(4.8) $$x^{2}+y^{2}=1.$$

Then the single-stance phase restarts by resetting the origin of the coordinate system to the new contact point. In this regard, despite the CoM’s motion being continuous, its x-coordinate experiences a discontinuity due to the origin shift,


(4.9) $$x^{+}=x^{-}-\lambda ,$$

where $x^{+}$ and $x^{-}$ are the *x*-coordinates of CoM just after toe-off and before it, respectively. The gait cycle ends when the stance leg re-stands vertically (x=0). Now, we can summarize all equations in a single Poincaré return map, which maps the states from *i*th mid-stance to (i+1)th mid-stance


(4.10) $$q_{i+1}=S(q_{i}),$$

where


(4.11) $$q=\{0,{\dot{x}}_{0},y_{0},{\dot{y}}_{0}\}.$$

At a fixed point that represents a limit cycle, we have


(4.12) $$q^{*}=S(q^{*}),$$

where


(4.13) $$q^{*}=\{0,{\dot{x}}^{*},y^{*},{\dot{y}}^{*}\}.$$

#### 4.1.2. Parameters and conditions for symmetric human-like limit cycle walking

We focus on symmetric limit cycle solutions. To this end, the first derivative of vGRF must be zero at mid-stance. So we have


(4.14) $$\dot{F_{y}}=0⟹\dot{y}=0\;\;\;\;at\;\;\;\;x=0.$$

As a result, the general form of initial conditions for equation (4.2) will be


(4.15) $$[x_{0},{\dot{x}}_{0},y_{0},{\dot{y}}_{0}]=[0,(1-\delta_{0})\Omega_{0},1-\delta_{0},0].$$

For limit cycles, there is a relation between $\delta_{0}$ and $\Omega_{0}$ to synchronize the radial displacement of the spring with its rotational movement, reducing the number of free parameters to be any three of the following four parameters:


(4.16) $$P=\{\lambda ,\gamma ,\Omega_{0},\delta_{0}\}.$$

In limit cycles with M-shaped vGRF and a maximum in height at the mid-stance, another constraint was applied, resulting in the following constraint $\ddot{F_{y}}\geq 0$ and $\ddot{y}\leq 0$, leading to the following inequality:


(4.17) $$\gamma \delta_{0}\leq (1-\Omega_{0}^{2}).$$

Using these equations and constraints, we found the limit cycles using standard techniques (see electronic supplementary material, appendix A.1).

### 4.2. Collection of walking data and fitting double spring-loaded inverted pendulum to walking data

The experimental data are collected from walking of a healthy subject (111 kg weight and 185 cm height) on a treadmill for one hundred steps at five different speeds, ranging from 1.5 to 3.5 mph, in increments of 0.5 mph, representing the slow, normal and fast walking of the subject. It is obtained based on the self-selected speed of the subject, followed by 20 and 40% slower and faster speeds. The GRFs were measured by force plates at 1000 Hz, and the hip coordinates were sampled by VICON at 200 Hz. Due to a high level of noise, we excluded data related to 1.5 mph from our analysis. The data were smoothed using the ‘smoothdata’ function in MATLAB, using the ‘sgolay’ method (Savitzky–Golay filter), which employs a quadratic polynomial fit to smooth data.

To assess DSLIP as a model for human walking, we employed two different strategies. First, we fit the model to each step separately, giving us an individual non-periodic trajectory for each step (optimized non-periodic trajectory). Second, by averaging empirical data for each walking speed, we fit a single limit cycle to all steps. The methods used to fit the walking data are described in the electronic supplementary material, methods (see electronic supplementary material, appendix A.2).

## Data Availability

Data are available at [48]. Supplementary material is available online [49].
